# Actual measurement, hygrothermal response experiment and growth prediction analysis of microbial contamination of central air conditioning system in Dalian, China

**DOI:** 10.1038/srep44190

**Published:** 2017-04-03

**Authors:** Yang Lv, Guangyao Hu, Chunyang Wang, Wenjie Yuan, Shanshan Wei, Jiaoqi Gao, Boyuan Wang, Fangchao Song

**Affiliations:** 1 School of Civil Engineering, Dalian University of Technology, Dalian, China; 2 School of Life Science and Biotechnology, Dalian University of Technology, Dalian, China; 3 China Academy of Building Research, Beijing, China; 4 Department of Biomedical and Chemical Engineering, Syracuse University, Syracuse, United States

## Abstract

The microbial contamination of central air conditioning system is one of the important factors that affect the indoor air quality. Actual measurement and analysis were carried out on microbial contamination in central air conditioning system at a venue in Dalian, China. Illumina miseq method was used and three fungal samples of two units were analysed by high throughput sequencing. Results showed that the predominant fungus in air conditioning unit A and B were *Candida* spp. and *Cladosporium* spp., and two fungus were further used in the hygrothermal response experiment. Based on the data of *Cladosporium* in hygrothermal response experiment, this paper used the logistic equation and the Gompertz equation to fit the growth predictive model of *Cladosporium* genera in different temperature and relative humidity conditions, and the square root model was fitted based on the two environmental factors. In addition, the models were carried on the analysis to verify the accuracy and feasibility of the established model equation.

With the large-scale use of central air conditioning system and the improvement of people’s living standard, more and more attention has been paid to the increasingly serious problem of indoor air pollution. Studies showed that air handing unit is an important source of microorganisms for indoor biological pollution, and some microorganisms tend to stay in the dust of air conditioning units with the appropriate temperature and humidity environment. The microorganisms grow and then enter the indoor space through the air, resulting in the destruction of indoor air quality^1^,^2^,^3^,^4^,^5^,^6^,^7^,^8^. National Institute for Occupational Safety and Health (NIOSH) conducted a study of 529 buildings, and the research results showed that among the sources of indoor air pollution, central air-conditioning system pollution accounted for 53%^9^. Summary of measurement by Professor Fanger showed that air-conditioning systems accounted for 42% in the indoor pollution sources^10^. Based on the supervision and inspection of air conditioning and ventilation system in the public places of China’s 30 provinces, municipalities and autonomous regions by China Academy of Building Research and the Chinese Center for Disease Control and Prevention, it was found that more than 90% of the central air conditioning systems could not meet China’s national sanitary standard^11^. Thus, air conditioning system should eliminate negative impact caused by its own, on this basis, it may relate to positive effect of the ventilation. In recent years, H1N1, SARS and other popular virus spread^12^,^13^, and some researches showed that the hygienic, reasonable air conditioning systems were important to reduce damage^14^,^15^. Therefore, microbial pollution in central air conditioning system has become a critical topic in the field of indoor air pollution.

Studies showed that the filter, cooling coil, humidifier, and cooling tower in central air-conditioning system were easy to microbial breeding^16^,^17^,^18^,^19^,^20^,^21^. In this study, a venue in Dalian was selected as the research object. As the working condition of the air conditioning system was down, the environment parameters were measured, and microorganisms existing on the wind pipe, filtering net, surface cooler, and condensate water, on the floor and in the air were collected. Besides, according to the tested microbial density and the identified genome sequence of collected microorganisms, the hygrothermal response experiment of dominant fungal was detected, and the fitting analysis was carried out based on the prediction model, followed by a series of statistical analysis. The aim of the present study was to clarify characteristics of the microorganisms in air conditioning systems, and the study would be helpful for policymakers and HVAC engineers to develop the appropriate strategies and to conduct the bacteria and fungi characteristic assessments in HVAC system.

## Measurement

### Preliminary survey

The object of study is a venue in Dalian, which covers a total area of 36400 m^2^ and building area is 17320 m^2^. The aboveground part includes a swimming pool, ball training venues, gymnasium, the lounge room and the clinic. The underground part consists of a table tennis hall, air conditioning equipment rooms and reservoir area. The whole building is centralized-controlled by the central air conditioning room, which includes two air handling units. Two measured units were all air system, which only had a coarse efficiency filter, and the unit is also provided with a heater, cooler and fan etc. Both units are the primary return air system and the filters are removable types. The running time of the air conditioning system is from May to October, and the daily operation period is 08:00–21:00. All components are cleaned every two years. When the measurement was carried on, the unit A and B were both cleaned a year ago. Both units were closed during the sample collection.

### Measurement Method

The actual measurement is divided into two parts: the environment parameter measurement and air unit sampling. First, the temperature, humidity, and CO_2_ concentration were automatically recorded by the temperature, humidity and CO_2_ monitor (MCH-383SD, Japan).

**Figure 1:**
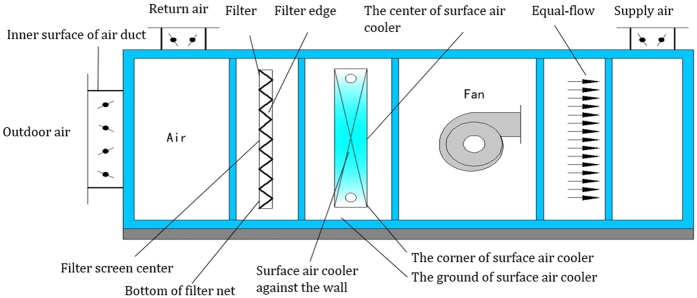
Composition diagram of air conditioning cabinet of gym (Unit A and Unit B).

Second, the disinfected planktonic microorganism sampler (HKM series DW-20, China) was installed where fresh air and return air mixed. Once installing the sampler, we loaded medium in the sampler and set the parameter of air flow in sampler as 2000 L loaded medium. After the sample collection, the Petri dishes must be sealed for preservation. Finally, according to the hygienic specification of central air conditioning ventilation system in public buildings of China^22^, we sampled the dust by using sterile non-woven (100 mm*100 mm) on components of unit A and B, respectively, and each sampling point covered a 15 cm*15 cm area at the sampling area. The non-woven fabrics were put into sterile water and stirred to make sure that organic substance on the non-woven was fully dissolved in sterile water. Then, the samples of sterile water containing organic substances were prepared in 10 times and 100 times diluted concentration, respectively. There are 10 sampling points in unit A and 5 points in B, and two measuring point positions of the units are shown in Fig. 1.

The Microorganisms collected in the air were directly cultured, and the samples in the dust were 10 times and 100 times diluted and 100 μL of the sample was inoculated into the two kinds of solid culture media. Beef extract peptone medium was used for cultivating bacteria and potato dextrose agar was used for cultivating fungus^22^,^23^. Each dilution was done in 3 parallel samples to reduce the error, and final results showed the average value. The blank samples and test samples were set up for each of the cultures. If there is no colony detected on the blank sample, the test results are valid.

Both field test and laboratory measurements were performed in accordance with the hygienic specification of central air conditioning ventilation system in public buildings requirements^22^.

### Genome Sequencing

Only a small part of microorganisms are cultivable. Therefore, the traditional cultivation method can not harvest all the species in ecological samples^24^. Fungal genome sequencing is an emerging method to identify the microbial genome, which could directly indicate related species information from environment samples^25^. Fungal amplicon sequencing analysis was used in this study, because the existing research showed that fungal spores have stronger vitality than other microorganisms in the air, and fungi dominated the microorganism in air conditioning systems. Therefore, this method was mainly used to identify fungi in this study^3^,^17^,^18^,^19^,^20^,^21^,^22^,^23^,^24^,^25^,^26^,^27^,^28^.

## Methods and Results

### Environment Parameters in Air Handling Units

Temperature, humidity and CO_2_ concentration of Unit A and B are shown in Table 1. Unit A is located in the ground floor (B1), and the unit B is located on the ground floor. Compared to the unit B, the humidity of unit A is higher, and the temperature is lower.

### Microbial Colony Analysis

**Figure 2:**
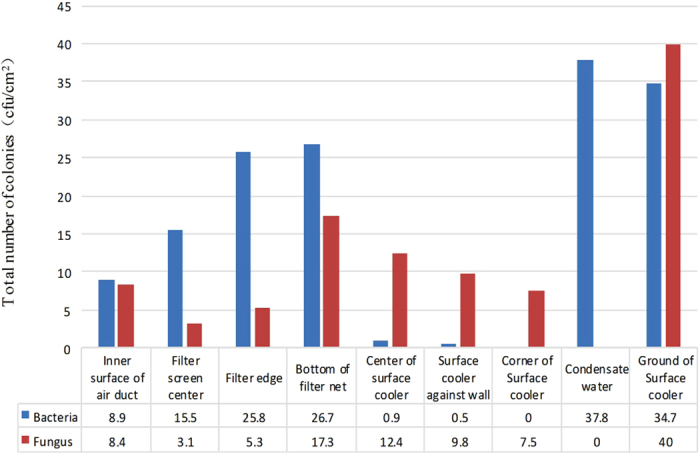
Statistics on the number of bacterial/fungus colonies in the unit A.

The distribution density of bacteria and fungi in the unit A is obtained through statistics, as shown in Fig. 2. The concentration of airborne fungus was 44 cfu/m^3^, and the concentration of airborne bacteria was 16 cfu/m^3^. The unit A showed the obvious microbial contamination status, though all components and airborne microorganism meet the Hygienic specification of central air conditioning ventilation system in public buildings of China^22^. The microbial distribution in filter net is central <edge <bottom and bacteria accounted for a larger proportion; the microbial distribution in surface cooler is center >against the wall >edge, and fungi accounted for a large. The fungal contamination in the air is more serious than the bacteria.

**Figure 3:**
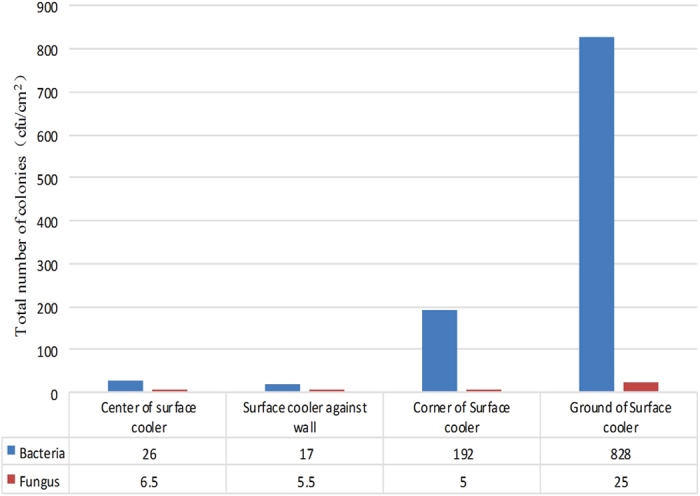
Statistics on the number of bacterial/fungus colonies in the unit B.

The distribution density of bacteria and fungi in the unit B were obtained through statistics, as shown in Fig. 3. The concentration of airborne fungus was 22 cfu/m^3^, and the concentration of airborne bacteria was 80 cfu/m^3^. Parts of the measuring point in the unit B were polluted seriously. The bacterial colonies in the corner and the ground of the surface cooler were beyond the hygienic index (≤100 cfu/cm^2^) in the Hygienic specification of central air conditioning ventilation system in public buildings of China regulates^22^. Limited by unit placement, there were less measuring points in unit B, and we chose the same measuring points in both units for comparison (centre of surface cooler, surface cooler against wall, corner of surface, and ground of surface cooler). The comparison between unit A and B indicates that the bacterial density in unit A was less than that in the same sampling point in unit B, but the fungal density in Unit A was more than that in the same sampling point in unit B.

If the cleaning and disinfection is not enough before the air conditioning system running, it may make the fungus to enter the indoor environment, which results in make the pollution of indoor air. Compared with cooling coil, the fungus contamination is worse in the floor dust and the air suspension. During the actual measurement, it is found that the unit internal is unprecedentedly narrow and low intensity of illumination in a closed state. According to the description by technicians, it is easy to trample damage to the underground pipes, which leads to the disinfection and cleaning work rarely in the unit.

### Fungal genome sequencing analysis

In this study, we analysed the samples from the sampling points A1, B1, and B2 by amplicon sequencing information analysis, respectively named A1A, B1A, and B2A.

All collected samples in the air conditioner were transferred to the Eppendorf tubes and processed with the extraction step. Samples were resusponded in TENS buffer with SDS and proteinase K as described by Vinod^29^. After incubation at 55 °C, phenol/chloroform/isoamyl alcohol was added to remove proteins, and the nucleic acid was precipitated with isopropanol and sodium acetate (0.3 M). Total DNA was dissolved in 1 × TE after washing with 75% ethanol. And then the quality and quantity tests were conducted by agarose gel electrophoresis, NanoDrop, and fluorometer (Qubit^®^ dsDNA High Sensitivity and dsDNA Broad Range assay, Life Technologies Corporation).

For Illumina MiSeq and HiSeq 2000 DNA sequencing, the qualified total DNA was used for preparation of amplicon libraries at the Beijing Genomics Institute (BGI, Shenzhen, China). For PCR product, the jagged ends of DNA fragment would be converted into blunt ends by using T4 DNA polymerase, Klenow Fragment and T4 Polynucleotide Kinase. Then add an ‘A’ base to each 3’ end to make it easier to add adapters. After all that, fragments too short would be removed by Ampure beads. For genomics DNA, we use fusion primer with dual index and adapters for PCR, fragments too short would be removed by Ampure beads too. In both cases, only the qualified library can be used for sequencing. The quality and quantity of libraries were assessed using the 2130 Bioanaylzer (Agilent Technologies) and the StepOnePlus Real-Time PCR System (Applied Biosystems).

The raw data generated by MiSeq and HiSeq 2000 sequencers was processed to eliminate the adapter pollution and low quality to obtain clean reads. The qualified clean data was used for the further bioinformatics analysis. Firstly, paired-end reads with overlap were merged to tags by software FLASH (v1.2.11)^30^, and then tags were clustered to OTU at 97% sequence similarity using USEARCH (v7.0.1090)^31^. Secondly, Taxonomic ranks were assigned to OTU representative sequence using Ribosomal Database Project (RDP) Na, e Bayesian Classifier v.2.2^32^. At last, alpha diversity, beta diversity and the different species screening were analyzed based on OTU and taxonomic ranks by MOTHUR (v1.31.2)^33^.

### Sequencing Data Statistics

In order to fully understand the community structure of fungal sample and analyse fungus microbial diversity, while excluding errors that human operation brings, genome sequencing method in fields of molecular biology was employed in this study to obtain micro biological information. Illumina Company developed Miseq method with higher flux and simple operation and lower cost for genome sequencing. Besides, the synthesis of side edge sequencing method with higher reliability is more suitable for laboratory community structure. The high-throughput sequencing was found to be useful to characterize compositions and diversities of moulds. The gene sequence of the test samples from genome sequencing was dealed with, such as stitching and matching, and the sample had a total of 59309 high quality fungal sequences, with an average length of 219 bp. The optimized sequence and the average length of the sample are shown in Table 2.

### OTU and Abundance analysis

**Figure 4:**
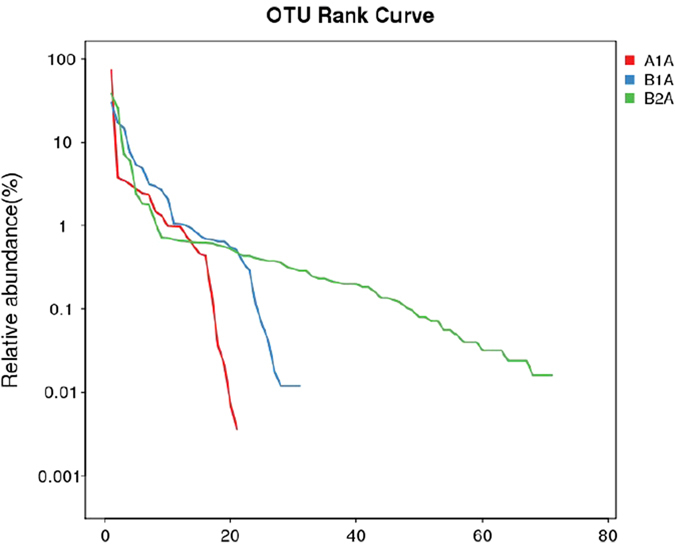
Rank OUT curve The horizontal coordinate for the sample OUT abundance ranking (from high to low), the longitudinal coordinates for the OTU abundance.

Stitching and optimising the tags, in order to be the OTU (Operational Taxonomic Units) for species classification, gathering in the 97% similarity, and the statistics of each sample in the abundance of information in each OTU were done^31^,^34^,^35^. Rank OTU curve is a form of species diversity in the sample, which can explain two aspects of sample diversity, that is, the richness and evenness of species in the sample. The richness of species in the samples represented by the horizontal length of the curve is wide, so that the sample species is more abundant. The uniformity of species in the samples from the curve reflects the longitudinal axis of the shape. That the curve is flat means that the sample has a higher composition of the species evenness. From Fig. 4, the species composition of B2A is the most abundant, and the uniformity is the highest.

### Sample diversity analysis of observed species

Alpha diversity is the analysis of species diversity in a single sample^33^, including the species observed index, Shannon index, etc. The greater the two indices are, the more abundant species is in the sample. The species observed index reflects the richness of the community in the sample, which also refers to the number of species in the community, without taking into account the abundance of each species in the community. Shannon index reflects the diversity of the community, and the species richness and evenness of species in the sample community. In the case of the same species richness, the greater the evenness of the species is in the community, the greater the diversity of the community is.

### Observed species exponential dilution curve

**Figure 5:**
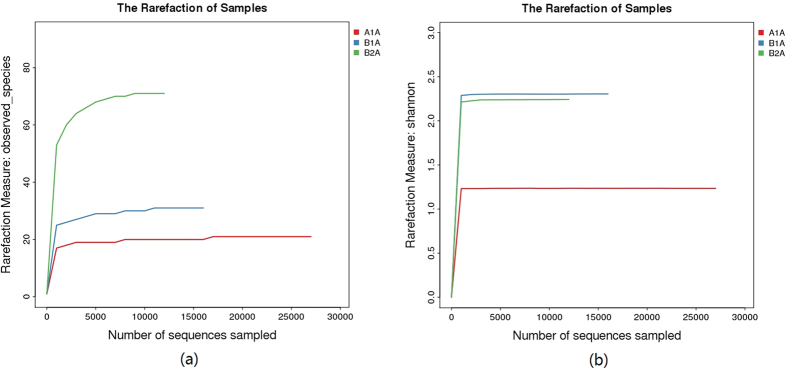
The dilution curve of observed species index (**a**) and shannon index (**b**).

Random sample in the processed sequence, draw the sequence number for the abscissa and the corresponding species number can be detected as the ordinate, so as to form a curve production dilution curve, shown in Fig. 5(a). With the increase of the sample quantity, the number of species increase and gradually become stabilized. When the curve reaches the plateau, it can be considered that the depth of the sequencing has been basically covered all the species in the sample. At the same time, the observed species index can reflect the richness of community in the sample, that is, the number of species in the community. It can be seen that the distribution of fungal species richness is B2A > B1A > A1A.

### Shannon exponential dilution curve

Shannon index is affected not only by species richness in the sample community, but also by the evenness of species. In the case of the same species richness, the greater the evenness of the species is in the community, the more abundant diversity of the community is. It can be seen in the Fig. 5(b) that the fungal species diversity of the unit B is significantly more complex than the unit A, and the similarity of species diversity in two sampling points of unit B was very high.

### Composition of microbial samples

**Figure 6:**
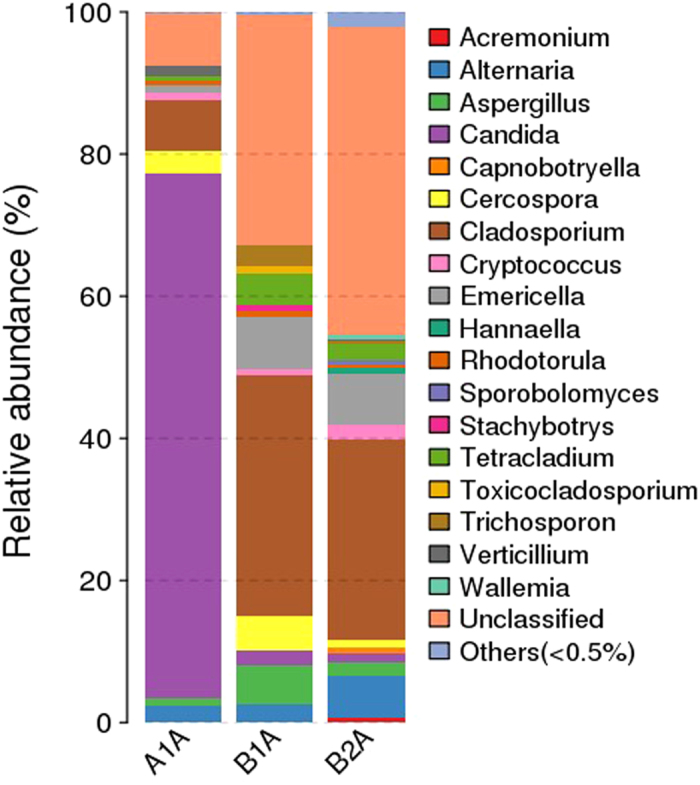
The taxonomic composition distribution in samples of Genus-level.

Figure 6 illustrates the species composition proportion of the three sampling points, and the proportion was redrew by removing the strains which were not detected in the sample. The results are shown in Table 3. The species with the largest proportion is the dominant fungi.

According to the fungal genome sequencing analysis results, fungal components in different units at the same sampling were different, and that in the same unit at different sampling points were roughly similar. They were caused by the different environmental conditions. On the center of air cooling coil in Unit A, *Candida* accounted for 80%; on the center and against the wall of the air cooling coil in Unit B, *Cladosporium* accounted for 50%, accompanied by *Alternaria, Emericella* and other fungus. *Cladosporium* is usually rich in outdoor air, but they will also grow on the indoor surfaces when the humidity is high. Existing research shows that the *Cladosporium* spore is an extremely important allergen in the airborne transmission, which could cause asthma attacks or similar respiratory disease in patients with allergic reactions^36^. Some species of *Candida* is a kind of conditional pathogenic fungi in the human body.

### Growth prediction analysis of models

Traditional microbial detection generally have the characteristics of hysteresis, which cannot play the role of prediction, but the use of mathematical models to predict the growth of microorganisms can timely and effectively predict the growth of microorganisms. Therefore, it is very important to study the growth prediction model of the fungi in the air conditioning system. According to environmental conditions mentioned before, we established growth kinetics prediction model of *Cladosporium* spp. to predict the rapid fungal growth in the experimental conditions, which can provide a theoretical basis for air microbial contamination prediction system and help evaluate the health risk inside buildings.

The models were fitted by Origin software (version 8) and Matlab R2014a, and the fitting conditions of Logistic model and Gompertz model were compared under different temperature and humidity conditions. The fitting effect between these two models and the fitting results of the two models were compared, and the corresponding model parameters were obtained. In addition, the square root model was fitted based on the two environmental factors.

### Experimental study on the hygrothermal response of fungus

**Figure 7:**
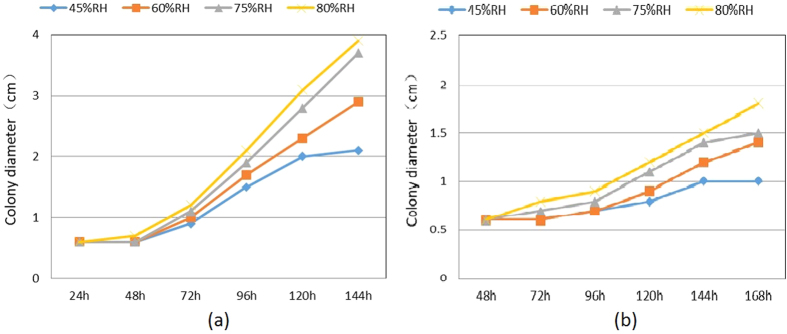
Changes of with the time of the colony diameter of *Cladosporium* (**a**) and *Penicillium* colonies (**b**) under different relative humidity conditions at 20 °C.

**Figure 8:**
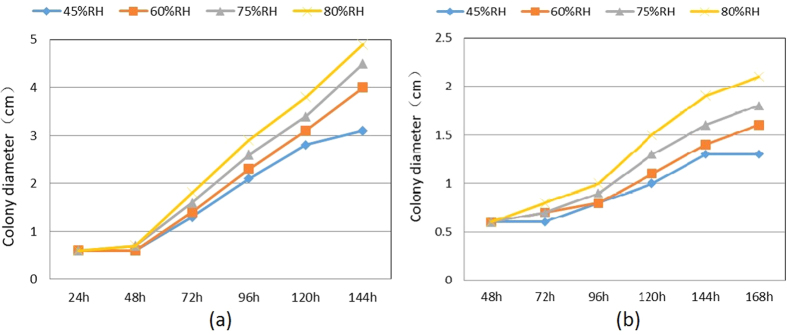
Changes of with the time of the colony diameter of *Cladosporium* (**a**) and *Penicillium* colonies (**b**) under different relative humidity conditions at 25 °C.

**Figure 9:**
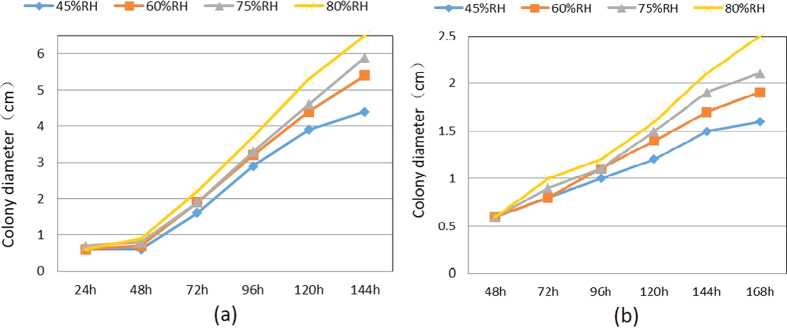
Changes of with the time of the colony diameter of *Cladosporium* (up) and *Penicillium* (down) under different relative humidity conditions at 30 °C.

Laboratory studies have revealed that fungal growth and reproduction are affected by water, temperature, nutrients, micro-elements, pH, light, carbon dioxide, and oxygen tension^37^.The most relevant determinants of fungal proliferation in the building context are water/moisture and temperature, and to a certain extent those factors affect other environmental factors such as substrate pH, osmolarity, nutrient, material properties etc^37^,^38^.In order to lay the foundation for the fitting model, and to study the growth characteristics of fungi in different temperature and relative humidity, we set an experimental study on the hygrothermal response of fungus. From the results of fungal genome sequencing and literature research^39^,^40^,^41^, we selected *Cladosporium* spp. and *Penicillium* spp. as the research objects which are both common in air conditioning systems.This paper mainly studied the status of microbial contamination in air handling units so that the air temperature of each part of the air handling unit should be considered. The temperature gradient of 20 °C −25 °C −30 °C and relative humidity gradient of 45%−60%−75%−80% were selected as experimental hygrothermal conditions. The results of hygrothermal experiments are shown in Figs 7, 8, 9. It can be known that growth rate of *Cladosporium* spp. is faster than that of *Penicillium* spp., in any experimental conditions, which is the essential characteristics of a strain, is hygrothermal response control method cannot change. These data indicated that low RH environments can reduce or even inhibit fungal growth. This observation agrees with findings by W. Tang and Pasanen^42^,^43^.

### Growth prediction analysis based on Logistic model

Logistic model is a typical model of biological ecology, which has been widely used in the field of biological ecology^44^. According to the actual research, the following formula equation (1) was obtained after the appropriate change of the Logistic equation. N was the colony growth diameter, cm; t was the microbial growth culture time, h; A_1_, A_2_, x_0_, P as the model parameters.


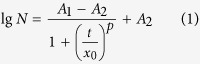


It can be seen from the Table 4, the fitting curve of Logistic model is similar to the experimental results. At 20 °C and 30 °C temperature conditions, the model’s fitting effect is excellent, and R^2^ is greater than 0.99; at 25 °C temperature conditions, the model fitting effect is not as good as other temperature conditions.

### Growth prediction analysis based on Gompertz model

Gompertz equation is the basic model for predicting the growth of microorganisms. The PMP program developed by the Ministry of agriculture to establish the model of pathogenic bacteria is the basic model for the study of Gompertz equation. Gompertz model has been widely used in the field of biology. Gompertz model expression was as equation (2):


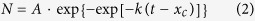


N was the colony growth diameter, cm; t was the microbial growth culture time, h; A, A, k, x_c_ as the model parameters.

It can be seen from the Table 5 that the fitting curve of Gompertz model is better fitted to the measured parameters. At the same temperature, with the increase of relative humidity, Gompertz model fitting effect is better; the model is well fitted at the temperature of 25 °C and the fitting effect is better than 20 °C and 30 °C temperature conditions. The fitting of Logistic model to the growth of the fungus is better than that of the Gompertz model. The two models are tested by the deviation factor B_f_ and the accuracy factor A_f_ in the mathematical model test. The accuracy and feasibility of the model are feasible and credible.

### The square root model fitting based on the two environmental factors

Temperature and relative humidity are the important factors affecting the growth of fungi, so it is necessary to study the influence of two environmental factors on the growth of fungi. In the establishment of prediction model of biological growth, if we need to consider the influence of various environmental factors, we must consider the number of variables and the independence between variables. The combined effects of water activity and temperature on the growth of *Staphylococcus xylosus* were studied by Mcmeekin^45^. They found that when T_min_ is fixed, for each φ, the relationship between growth rate and temperature can be described by using the square root model. The combined effects of these two variables can be expressed by the modified equation (3):


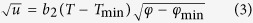


In the formula, u is the growth rate of fungus, cm/h; b_2_ is the coefficient; T is the culture temperature,  °C; T_min_ is the most important parameter of square root equation, and it refers to the lowest temperature when the growth rate is zero, °C; φ is relative humidity of the cultivation, %. By using the Logistic primary model, the predictive value of the growth rate of the *Cladosporium* colony growth rate (instantaneous velocity) was obtained, as Table 6 shows.

**Figure 10:**
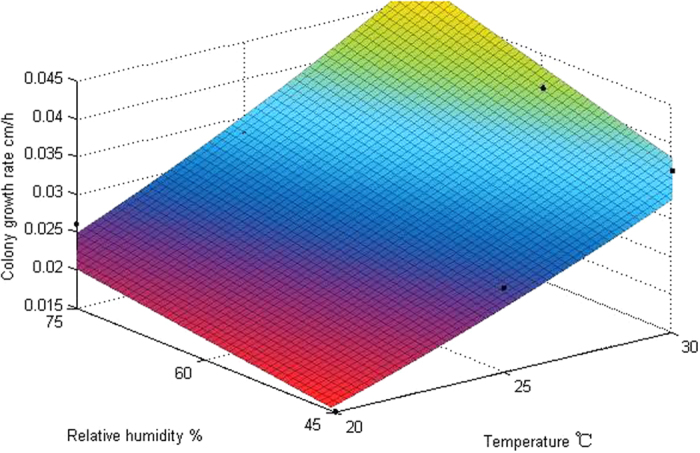
Model fitting of predicting growth of *Cladosporium* in double factor square root model.

Through the model fitting, the parameters of the square root model could be obtained, as Table 7 shows, and the model fitting of predicting growth of *Cladosporium* was shown as Fig. 10. The model equation of B_f_ value was between 0.90–1.05, indicating that the model used to predict the range of the experimental environment in *Cladosporium* colony growth condition. At the same time, the A_f_ value of the model was 1.05169 that is closed to 1, which shows that the model has high accuracy.

## Conclusions and Suggestion

This study selected two central air conditioning systems at a venue in Dalian as the objects. Actual measurement and a series of studies were carried out on microbial pollution characteristic, and the results are shown as below:

1. The bacterial colony forming units of the two measuring points in unit B were 192 cfu/cm^2^ and 828 cfu/cm^2^, respectively, which exceeded the hygienic specification of central air conditioning ventilation system in public buildings of China (≤100cfu/cm^2^), and the rest of the test points met the relevant standards of China. The distribution of bacteria was more than fungi, and the concentration was higher. With the total characteristics of different distribution density, the area of dust associated microorganisms and the air pollution were more serious.
2. *Alternaria* spp., *Candida* spp., *Cercospora* spp. and *Cladosporium* spp. existed in both units. The *Candida* spp. accounted for 80% in Unit A, and the *Cladosporium* spp. occupied 50% in Unit B. The composition of fungi in B was more complicated. Two dominant fungi are both deleterious to health, so the timely maintenance and cleaning are required. It is suggested that the operating space should be reserved in the air conditioning room, so as to avoid incomplete cleaning and disinfection.
3. Within the experimental temperature and relative humidity, with the increase of relative humidity or temperature, the colony growth of the same strain showed an increasing trend. For the prediction model of the fungus growth, the study found that the overall fitting effect of Logistic model is better, and R^2^ values were greater than 0.97. Logistic model for the *Cladosporium* spp. growth was better than Gompertz model. At the same time, considering the influence of temperature and relative humidity, the square root model can well predict the growth of *Cladosporium* spp. It provides a theoretical basis for the growth of fungi in the air conditioning system under the hygrothermal environment conditions.

## Additional Information

**How to cite this article:** Lv, Y. *et al*. Actual measurement, hygrothermal response experiment and growth prediction analysis of microbial contamination of central air conditioning system in Dalian, China. *Sci. Rep.* **7**, 44190; doi: 10.1038/srep44190 (2017).

**Publisher's note:** Springer Nature remains neutral with regard to jurisdictional claims in published maps and institutional affiliations.

## Figures and Tables

**Table 1:** Environment parameters of two units.

Condition	Unit A	Unit B
	Closed	Closed
Temperature (°C)	23.7	25
Relative Humidity (%)	84.4	70.6
CO_2_ Concentration (ppm)	2282	1826

**Table 2:** Sample sequence statistics.

Sample name	Sequence number	Base number (bp)	Average length (bp)
A1A	28326	6146742	217
B1A	17705	3912805	221
B2A	13278	2921160	220

**Table 3:** Species composition proportion (N means nothing).

Fungus strains	A1A (%)	B1A (%)	B2A (%)
*Alternaria*	2.53	3.96	11.02
*Aspergillus*	N	7.95	3.34
*Candida*	80.08	3.10	2.51
*Cercospora*	3.40	7.26	2.01
*Cladosporium*	7.86	50.48	51.57
*Cryptococcus*	N	N	4.22
*Emericella*	N	11.07	13.12
*Tetracladium*	N	6.61	4.45
*Trichosporon*	N	4.44	N
Others	6.14	5.11	7.75

**Table 4:** Model fitting and model parameters of Logistic.

Temperature	RH	A_1_	A_2_	x_0_	p	R^2^
20 °C	45%	0.5710	2.7853	104.0955	4.6632	0.9968
	60%	0.5523	3.7504	112.7345	4.01763	0.9946
	75%	0.5425	5.5873	126.0084	3.78028	0.9970
25 °C	45%	0.4964	5.9770	130.1226	3.1022	0.9869
	60%	0.4932	5.7732	118.4491	3.3263	0.9888
	75%	0.4684	7.6131	132.7818	2.8325	0.9876
30 °C	45%	0.5299	5.3939	93.6220	5.0198	0.9978
	60%	0.4840	6.9530	105.2175	3.5773	0.9943
	75%	0.58170	8.4542	116.9170	3.4122	0.9951

**Table 5:** Model fitting and model parameters of Gompertz.

Temperature	RH	A	x_c_	k	R^2^
20 °C	45%	13.5489	237.7993	0.0057	0.9628
	60%	16.1259	228.8224	0.0063	0.9749
	75%	29.4803	263.1720	0.0061	0.9840
25 °C	45%	12.6735	168.2450	0.0085	0.9833
	60%	9.8060	133.2720	0.0107	0.9830
	75%	10.5684	129.8359	0.011	0.9875
30 °C	45%	6.6530	85.0694	0.0214	0.9732
	60%	8.5414	96.1094	0.0166	0.9879
	75%	12.2978	119.2325	0.0128	0.9891

**Table 6:** The measured value of the growth rate of *Cladosporium* in Logistic model.

Relative humidity	20 °C	25 °C	30 °C
45%	0.0151	0.0262	0.0363
60%	0.0193	0.0287	0.0404
75%	0.0261	0.0327	0.0437

**Table 7:** Model fitting and model parameters of double factor square root.

b_2_	T_min_	φ_min_	R^2^
0.00521	−4.89647	−0.60914	0.95658
